# Tumour Cell Heterogeneity

**DOI:** 10.12688/f1000research.7210.1

**Published:** 2016-02-29

**Authors:** Laura Gay, Ann-Marie Baker, Trevor A. Graham

**Affiliations:** 1 Evolution and Cancer Laboratory, Centre for Tumour Biology, Barts Cancer Institute, Barts and the London School of Medicine and Dentistry, Queen Mary University of London, London, EC1M 6BQ, UK

**Keywords:** Cancer evolution, intra-tumour heterogeneity, evolutionary biomarkers, personalised medicine

## Abstract

The population of cells that make up a cancer are manifestly heterogeneous at the genetic, epigenetic, and phenotypic levels. In this mini-review, we summarise the extent of intra-tumour heterogeneity (ITH) across human malignancies, review the mechanisms that are responsible for generating and maintaining ITH, and discuss the ramifications and opportunities that ITH presents for cancer prognostication and treatment.

## The origins of intra-tumour heterogeneity

Intra-tumour heterogeneity (ITH) has been documented for many decades, initially from a morphological perspective ^1, 2^. Cancers of all types are now recognised to consist of highly diverse populations of cells ^3^, where ITH is detectable at the genetic, epigenetic, and phenotypic levels (see Table 1 for a pan-cancer summary). Recent advances in next-generation sequencing and microarray technology have enabled researchers to begin to appreciate the full extent and complexity of ITH. As a major cause of targeted therapy failure and disease resistance ^4^, ITH is a subject of much biological and clinical relevance.

**Table 1.** Summary of a selection of studies revealing intra-tumour heterogeneity in space and time.

Tissue type	Selected references	Summary of measured intra-tumour heterogeneity
Kidney	Gerlinger *et al*. ^8^ (2012)	Spatial genetic intra-tumour heterogeneity (ITH) measured by multi-region whole exome sequencing (WES) and/or single-nucleotide polymorphism (SNP) array analysis of four cases of renal-cell carcinoma and associated metastases. Phenotypic ITH was established by using immunohistochemistry and mRNA expression profiling. Reported extensive ITH and branched tumour evolution.
	Gerlinger *et al*. ^9^ (2014)	Spatial genetic ITH measured from multi-region WES and ultra-deep targeted sequencing in 10 clear cell renal carcinomas; 67% of non-synonymous somatic mutations identified were heterogeneous between sampling sites. Increasing the number of biopsies analysed increased the extent of ITH identified. Intra-regional (within-biopsy) subclonal structure was identified on comparison of variant allele frequencies (VAFs) of the genetic changes present.
Lung	de Bruin *et al*. ^10^	Spatial genetic ITH measured from multi-region WES and whole genome sequencing (WGS) from seven non-small cell lung cancers. A subclonal structure was identified between sampled regions, and intra-regional diversity was measured by using VAFs. Assembly of phylogenetic trees allowed temporal dissection of the heterogeneity in the type of genetic events; the majority of mutations in driver genes were identified as early events.
	Zhang *et al*. ^11^	Spatial genetic ITH measured from multi-region WES in 11 lung adenocarcinomas. ITH was identified between and within regions analysed. Patients who relapsed after surgery had a significantly larger proportion of subclonal mutations in the primary tumour than those who did not; therefore, higher ITH may be related to relapse.
Colon	Dalerba *et al*. ^12^	Phenotypic ITH measured from single-cell polymerase chain reaction gene expression analysis. The expression profiles from monoclonal tumour xenografts (implantation of a single cell) recreated the heterogeneity of the cellular composition of the primary tumour, demonstrating that transcriptional diversity in colon cancer can be explained by multi-lineage differentiation and not purely by clonal genetic heterogeneity.
	Kim *et al*. ^13^	Spatial genetic ITH measured from multi-region WES and comparative genomic hybridisation arrays in five primary tumours and associated liver metastases; 50–80% of all mutations identified were subclonal. There were regional differences in the prevalence of mutation spectra and other aberrations (notably, regional chromothripsis). Phylogenetic analysis identified branching evolution during progression, with pre-existing subclones in certain regions of the primary lesions related to the metastasis.
	Sottoriva *et al*. ^14^	Spatial genetic ITH measured from genomic profiling (WES, targeted deep sequencing, SNP arrays, fluorescent *in situ* hybridisation [FISH], and neutral methylation tag sequencing) of 349 glands sampled from opposite sides of 15 primary lesions. The pattern of ITH was used to infer the mechanism of early tumour growth. ITH was uniformly high, with subclone mixing (variegation) in glands from distant regions and lack of evidence for recent clonal selective sweeps, suggestive of a “big bang” whereby tumours grow in a single expansion at an early stage in development, scattering the early intermixed clones.
	Kreso *et al*. ^15^	Phenotypic ITH, measured by proliferation, survival, and chemotherapy response, identified by serial xenotransplantation of spatially distinct tumour regions.
Brain	Snuderl *et al*. ^16^	Genotypic ITH using FISH to identify receptor tyrosine kinase (RTK) amplifications in archival glioblastoma (GBM) samples. Mosaic amplification of up to three different RTKs was observed in cells of the same tumour in a mutually exclusive fashion, indicating coexisting subpopulations. These cells shared other genetic events in *TP53* or *CDKN2A*, signifying that they originated from the same precursor.
	Sottoriva *et al*. ^17^	Spatial genetic ITH measured from multi-region genome-wide copy number alterations (CNAs) from 11 GBMs. Reported extensive ITH and used phylogenetic analysis to infer early (clonal), intermediate (subclonal), and late (unique) events. Cellular phenotypic ITH measured from multi-region gene expression microarrays identified heterogeneity in phenotypic subtypes, often with more than one coexisting within the same tumour. Epigenetic ITH was analysed within each tumour region on the single-molecule level by using neutral methylation loci, with no single dominant clone identified in any region of the tumour analysed.
	Johnson *et al*. ^18^	Temporal genetic ITH measured from WES of 23 GBMs and recurrences; only half of the mutations detected in primary tumour were also identified in the recurrence. Genetic ITH in response to temozolomide therapy was also examined in these samples with hypermutation and notable alkylation damage mutation signatures in the recurrences.
	Patel *et al*. ^19^	Phenotypic ITH measured from RNA sequencing of 430 single cells sorted from five primary GBMs. Extensive ITH was demonstrated at the transcriptional level, in particular for RTKs. Although each tumour had a dominant phenotypic subtype of GBM, subsets of cells within the same tumour were found to express alternative phenotypic subtypes, and heterogeneity in subtype was associated with decreased patient survival.
	Meyer *et al*. ^20^	Phenotypic ITH measured from 44 single cell-derived clones from four primary GBMs. Cells were selected by using fluorescent-activated cell sorting for stem cell markers to enhance for clonogenic activity. Clones from the same tumour showed variable protein expression of key drivers phosphatase and tensin homolog (PTEN) and epidermal growth factor receptor, and wide variability in proliferation and differentiation abilities, and response to therapies. The variable treatment response of clones correlated with transcriptional clonal heterogeneity as assessed by mRNA microarray. Genetic ITH of clones was assessed using SNP arrays, and CNAs in genes/ pathways that associated with the phenotype were observed in the clones.
Blood Acute lymphoblastic leukaemia (ALL)	Mullighan *et al*. ^21^	Changes in genetic ITH over time in response to treatment measured by SNP array in 61 primary tumour-relapse sample pairs. In more than 90% of cases, there was a marked change in the pattern of CNAs between diagnosis and relapse, with CNAs acquired in the relapse often affecting cell cycle regulation and B-cell development. The diagnosis and relapse samples nearly always had a common clonal origin, but cells responsible for the relapse were present as a minor subclone in the diagnostic sample.
	Anderson *et al*. ^22^	Genetic ITH measured in 30 cases by using single-cell multiplex FISH with probes for common gene fusions and CNAs. ALLs were found to have a complex subclonal architecture and branching evolution. The same CNAs reoccurred in different subclones independently and in no preferential order. Temporal genetic ITH was observed between pre-leukaemic aplasia and ALL at diagnosis, as well as between diagnosis and relapse, with dynamic shifts in subclonal dominance.
Acute myeloid leukaemia (AML)	Ding *et al*. ^23^	Temporal genetic ITH in response to chemotherapy measured by WGS and targeted deep sequencing of eight primary tumour-relapse pairs. VAFs were used to estimate clonal population size. Two clonal evolution patterns were identified in response to treatment: (1) acquisition of new mutations in the founding clone enabling it to evolve into the relapse clone and (2) an evolutionary bottleneck occurs, with eradication of all of the major subclones of the founding clone, except one. The remaining subclone gains additional mutations and expands at relapse.
	Walter *et al*. ^24^	Temporal genetic ITH measured by WGS and targeted deep sequencing in seven paired bone marrow samples from patients with secondary AML and the preceding myelodysplastic syndrome stage. In all cases, the founding clone progressed to acute leukaemia by acquiring many new mutations; there was emergence of a new subclone in some cases.
Lymphoma	Okosun *et al*. ^25^	Temporal genetic ITH measured by WES/WGS in 10 follicular lymphoma cases up to, and including, transformation. Construction of phylogenetic trees from VAFs identified multiple subclones and a branching pattern of evolution. The majority of transformed samples shared many trunkal mutations with the untransformed samples; however, in rare cases, the transformed and untransformed clones shared very few mutations, indicating earlier divergence.
Prostate	Brocks *et al*. ^26^	Epigenetic and genetic ITH measured by analysis of DNA methylation and CNAs, respectively, in multi-region samples (primary tumour, premalignant lesion, and lymph node metastasis) from five patients. Extensive variability was apparent at DNA methylation enhancer sites. Multiple subclonal populations were identified from both the DNA methylation and CNA datasets. There was a close resemblance in the structure of phylogenetic trees constructed from the epigenetic and genetic data, indicating a similarity in evolutionary processes.
	Boutros *et al*. ^27^	Spatial genetic ITH measured by WGS from multiple archival biopsies of five localised multi-focal cancers. Tumours were found to be highly heterogeneous in single-nucleotide variants (SNVs) and CNAs between sampling sites, with evidence for divergent tumour evolution.
	Cooper *et al*. ^28^	Spatial genetic ITH measured from WGS, targeted deep sequencing, and FISH of ERG alterations in multiple samples from three multi-focal prostate cancers and surrounding normal tissue. Clonal expansions/fields were identified in normal tissue, and some of the field genetic changes also were present in areas of the tumour. The field effect in normal tissue may explain the branching phylogenies and clone mixing observed in the tumours.
	Gundem *et al*. ^29^	Temporal and genetic ITH measured from WGS in 10 primary tumours and multiple subsequent metastases that developed after androgen-deprivation therapy. Examination of clonal relationships between metastatic samples identified groups of subclonal mutations across multiple metastases, suggesting polyclonal seeding between different sites.
Breast	Park *et al*. ^30^	Phenotypic and genetic ITH measured from immunofluorescence staining and FISH (for common CNAs) in 15 invasive breast tumours, containing both *in situ* and invasive subregions within the same tissue section. There was a high degree of intra-tumour variability in the expression of markers for stem-like cells (CD44 ^+^) and more differentiated cells (CD24 ^+^). There was also a high degree of genetic heterogeneity both within and between these distinct tumour cell populations.
	Navin *et al*. ^31^	Spatial genetic ITH measured from single-nucleus sequencing in 200 cells taken from different geographical areas of two triple-negative ductal carcinomas and one paired metastatic liver carcinoma. Copy number profiles were used to elucidate differences in tumour subclone structure and evolution.
	Nik-Zainal *et al*. ^32^	Genetic ITH measured using high-depth WGS data from single bulk samples taken from 21 breast cancers. Subclonal diversity was a prominent feature with many mutations present in only a small amount of cells; however, all tumours contained a dominant subclone (>50% cells). Mutational processes were heterogeneous throughout cancer development.
	Wang *et al*. ^33^	Spatial genetic ITH measured from multiple single-nucleus WGS, WES, and copy number profiling to define clonal diversity in an oestrogen receptor (ER)-positive and a triple-negative carcinoma. No two single tumour cells were found to be genetically identical, and a large number of subclonal and unique mutations were identified. Single-molecule duplex sequencing estimated that many diverse mutations occurred at low VAF within the tumour.
Ovary	Khalique *et al*. ^34^ (2007)	Spatial genetic ITH of 16 cases of untreated high-grade serous ovarian cancer (HGSOC) measured by multi-region microsatellite and SNP analysis. Reported extensive ITH in all cases.
	Khalique *et al*. ^35^ (2009)	Spatial and temporal genetic ITH measured by multi-region microsatellite analysis in 22 cases of untreated, metastatic HGSOC. Analysis of loss of heterozygosity (LOH) values revealed that ITH in metastases was less than primary tumours, although this was not statistically significant. Phylogenetic analysis revealed that metastases are clonally related to the primary tumour; however, the metastatic clone may have arisen at an early or late stage in the evolution of the tumour.
	Bashashati *et al*. ^36^	Spatial and temporal genetic ITH measured by multi-region SNP array and WES of 31 samples from six patients with untreated HGSOC. Phenotypic ITH measured by multi-region gene expression profiling. Revealed the high diversity of evolutionary trajectories displayed in HGSOC prior to treatment intervention.
	Schwarz *et al*. ^37^	Spatial and temporal genetic ITH measured by SNP array copy number profiling and selected WGS of 135 samples from 14 patients with HGSOC who received platinum-based chemotherapy. Patients who displayed a higher ITH had shorter progression-free and overall survival.
Premalignant disease		
Colonic adenomas	Novelli *et al*. ^38^	Spatial genetic ITH in microadenomas assessed by X/Y chromosome FISH in a sex chromosome mixoploid mosaic (XO/XY) patient with familial adenomatous polyposis (FAP). Areas of excised microadenomas were of mixed XO/XY genotype, indicating polyclonality in tumour origin.
	Thirwell *et al*. ^39^	Spatial genetic ITH measured in multiple individual crypts from 10 FAP microadenomas. Analysis revealed two clones carrying different somatic adenomatous polyposis coil ( *APC*) mutations in addition to the founding *APC* mutation, therefore indicating a polyclonal origin. Phylogenetic analysis using limited genetic markers ( *APC*, *KRAS*, and *TP53* mutations; LOH of 5p, 17p, and 18q) in 11 sporadic carcinoma-in-adenomas revealed different subclones between regions of carcinoma and low- and high-grade dysplasia.
Barrett’s oesophagus	Maley *et al*. ^40^	Spatial genetic ITH measured in 268 cases; biopsies were sampled every 1–2 cm along the Barrett’s segment, and genetic diversity (number of clones and genetic divergence) was calculated in each sample by measuring for aberrant DNA ploidy, LOH, microsatellite instability, and *CDKN2A* or *TP53* mutations. Barrett’s segments with greater clonal diversity were more likely to progress to cancer.
	Leedham *et al*. ^41^	Spatial genetic ITH measured in 164 individual glands that were laser capture-microdissected from 16 samples of eight Barrett’s oesophagus cases. Glands were screened for tumour suppressor gene loss of heterozygosity (LOH) and *CDKN2A*/ *TP53* mutations. Marked heterogeneity between glands was identified across individual samples, and multiple independent clones were present (bearing no shared founder mutation between the clones). A mosaic pattern of clones across the Barrett’s segment was observed.
	Li *et al*. ^42^	Spatial and temporal genetic ITH assessed by SNP array in samples from 79 Barrett’s oesophagus cases that had progressed to oesophageal cancer and 169 non-progressors. Samples from two time points (mean of 8.6 years apart) were evaluated per case, and biopsies were taken at every 2 cm of the Barrett’s segment at each sampling. The non-progressor genomes contained a small number of limited CNA events that had typically expanded throughout the Barrett’s segment and then remained stable over time. In contrast, the progressors developed significant genomic diversity as they approached cancer diagnosis.

When viewed through the lens of evolutionary biology, the sometimes extreme levels of diversity present in cancers ^5^ should come as no surprise ^6^. Carcinogenesis is an evolutionary process whereby somatic cells acquire random (epi)mutations that alter their phenotype, and the fittest new clones clonally expand because of the action of Darwinian natural selection ^7^; repeated rounds of mutation and natural selection can lead to the development of a malignant cancer clone that is capable of migration and growth in remote sites. Diversity in the evolving cancer ecosystem is inevitable because it fuels the evolutionary fire; there can be no “survival of the fittest” if all the cells in the tumour have the same fitness.

There are many mechanisms that contribute to ITH, and these can be broadly classified as “cell autonomous” or “non-cell autonomous”. An example of a cell-autonomous mechanism is the persistence of small numbers of errors that occur during DNA replication ^43, 44^. When multiplied by the billions of cell divisions required to produce even the smallest clinically detectable tumour (with a volume of approximately 1 cm ^3^), this low level of mutation can potentially generate tremendous within-tumour genetic diversity. Moreover, the rate at which diversity is generated in a tumour is typically accelerated by genetic instability, likely a consequence of replication stress ^45^, and the “mutator phenotype” ^46^ that is a feature of most solid tumours. Furthermore, there can be rare but catastrophic DNA replication errors occurring during a single mitosis that can lead to the production of daughter cells with grossly altered genomes ^32, 47, 48^.

A result of recent advances in next-generation sequencing is that single-cell whole genome sequencing is now possible (and indeed transcriptome sequencing too) ^49^. Therefore, it is conceivable that we will soon be able to envisage mapping the genetic diversity of an entire tumour at cellular resolution. However, an important question is how much of this information about genetic ITH will prove to be clinically relevant? Intriguingly, in some cancers (lung ^10, 11^ and colon ^13, 14^), the key driver mutations are proven to be clonal (e.g. present in all tumour cells), although spatially localised drivers have been found in other cancer types (e.g. kidney ^9^). The obvious question then becomes how many, if indeed any, of the heterogeneous mutations are important for tumour growth? Clearly, there is a need to discriminate between ITH that is attributable to “mutational noise” (e.g. the background mutation rate) and that which is in some sense “functional” for tumour development. Mathematical modelling of the amount of ITH that should be expected in a growing tumour can be helpful here, as an increase or decrease of ITH compared with expectation reveals “important” evolution in the tumour (our effort to implement such a model ^50^ is discussed briefly below). An alternative empirical approach would require the concurrent measurement of genotype and phenotype so that genetic ITH can be related to the presence of (minority) cell populations with biologically distinct function. Interestingly, measurement of the behaviour of different clones within a colon cancer, including their sensitivity to cytotoxic drugs, revealed marked differences in behaviour between tumour regions, without concomitant differences in genotype ^15^. This study in particular highlights how genetic ITH can be a poor proxy for functional heterogeneity, and the latter is clearly of greater clinical relevance, especially ITH of drug response.

A further source of ITH is the persistence of a cancer stem cell (CSC) hierarchy, a factor that may be described as either cell-autonomous (i.e. “stemness” is governed by a cell’s genetic or epigenetic makeup) and non-cell autonomous (i.e. “stemness” is governed by external factors within an environmental “niche”). Although a strict hierarchy of differentiation is described as a feature of haematopoietic cancers ^51^, a form of CSC architecture may significantly contribute to ITH in solid tumours. There is evidence of a CSC hierarchy in mouse models of brain cancer ^52, 53^, where a small subpopulation of cells is responsible for sustaining tumour growth. The definition of a CSC continues to be debated ^54^, but if CSCs are defined simply as the population of cells with long-term self-renewal capability, then following debulking chemotherapy tumour regrowth is determined by the prevalence of the CSC phenotype amongst the surviving cells ^18^. The question of the plasticity of the stem cell phenotype is an interesting topic of discussion. It is noteworthy that observations of cell populations growing *in vitro* ^55^ and genetically engineered mouse models of intestinal tumourigenesis ^56^ suggest that bidirectional phenotype switching can occur (even in the absence of clonal selection), questioning whether the “stemness” of a tumour cell is strictly intrinsically defined. Plasticity of a CSC phenotype can be viewed as a form of epigenetic heterogeneity within the tumour.

Heterogeneity in the microenvironment of a cancer can be described as a non-cell autonomous driver of cancer cell diversity ^62^; in a highly diverse microenvironment, different cellular phenotypes may be selected for or against in different regions of the tumour. For example, any sizeable tumour will inevitably contain areas of hypoxia and normoxia. In a hypoxic region, an anaerobically metabolising cell is expected to have a fitness advantage over an aerobically metabolising cell and so should repopulate the hypoxic region, but the opposite should be true in a normoxic region ^63^. Such different phenotypes may be genetically determined (such as the case of VHL/HIF1 mutants in kidney cancer) ^64^, or they may be a consequence of plasticity in cellular behaviours ^15^, and furthermore there may be feedback between the tumour cells and their microenvironment that drives specialisation of tumour cells and the concomitant strengthening of microenvironmental gradients.

A further example of a non-cell autonomous driver of ITH is the interactions between subclones of the tumour. In experimental systems, synergistic or predatory interactions between phenotypically distinct clones drive tumour growth ^15, 58, 59, 65– 67^. Intermixing of clonally distinct populations is a feature of many tumours and has been particularly well documented in gliomas ^16^. If interactions between cells within a tumour are critical for tumour maintenance, then reducing the tumour population size below some critical threshold—called an Allee threshold—may be an effective cancer treatment ^61^. The logic behind this idea is that small populations are not able to produce sufficient density of cooperative factors that are necessary for tumour maintenance (such as diffusible growth factors) and so small populations are unviable. The interplay between clones that are *producers* (e.g. angiogenic cells) versus *consumers* (e.g. aerobically respiring non-angiogenic cells) should, theoretically, also influence phenotypic diversity within a cancer. Clearly, tumour cells can also benefit from factors produced by the stromal cells in their microenvironment ^68^. *Clonal interference* refers to the situation where two or more clones of similar fitness each impede the growth of the other by competing equally well for limited resources, and theoretical models of cancer growth predict that clonal interference should slow cancer evolution and lead to the longer-term maintenance of multiple distinct clonal populations ^69^. Interactions between tumour cells and the immune system also shape ITH: for example, the immune system predates the tumour cells, and tumour cells that have evolved to avoid immune detection will persist and perhaps clonally expand in the tumour ^70^.

Recognising that there is a dynamic interplay between tumour cells and their microenvironment, and between tumour cells themselves, means that cancers are best viewed as complex evolving *ecosystems* ^60^. In the cancer ecosystem, the relative fitness conferred by a new mutation is defined by microenvironmental context ^6, 57^, where the context refers to both the neighbourhood of tumour and stromal cells and more broadly diffusible factors. Importantly, the ecological viewpoint provides a single framework to understand the seemingly distinct contributions of cell-autonomous and non-cell autonomous factors to ITH. This is because the ecological view forces us to recognise that all the evolution within a tumour is only ever driven by selection in the current microenvironment context: cell-autonomous drivers provide an advantage to the cell in their current context, and non-cell autonomous factors can drive evolution by changing that context.

Multi-faceted networks are challenging to understand, and consequently computational or mathematical models are increasingly recognised as essential tools to integrate and interpret the complex, multi-scale data derived from the interplay between tumour subclones and their interactions with the microenvironment (ref. 71 provides an overview). Part of the value of mathematical models is the ability to elucidate the underlying (perhaps simple) causes of intricate patterns in complex systems. For example, our own work has used a simple mathematical model of mutation during the first few rounds of cell doubling at the start of tumour growth to explain the complex pattern of genetic ITH observed in cancer ^50^. We note that this kind of approach can help to delineate “important” ITH from the inconsequential “mutational noise” mentioned above. More generally, such models can be used to generate new hypotheses concerning the generation and maintenance of ITH, and the effects of ITH on tumour evolution, and to predict response to therapy and recurrence.

In summary, the fact that tumours are an evolving ecosystem means that ITH is inevitable, and consequently ITH is observed in all tumour types and premalignant diseases where it has been looked for. We devote the remainder of this review to understanding the consequences of ITH for cancer prognostication and treatment.

## Intra-tumour heterogeneity and prognostication

Traditionally, cancer prognosis has been determined by the presence or absence of a particular feature within a tumour. Histopathology remains the mainstay of this approach: tumours are scored for stage (how far the cancer has invaded) and grade (a measure of how abnormal the cancer cells appear and how disrupted the tissue architecture of the tumour is compared with normal tissue). Molecular markers have entered clinical practice too, most prominently assessment of oestrogen receptor (ER), progesterone receptor (PR), and HER2 status in breast cancer ^73, 74^ (to determine prognosis and treatment choice). Relatedly, genetic analysis is employed to predict the likely efficacy of targeted therapies by testing whether the tumour contains pre-existing resistant clones; for example, colorectal cancers are screened for *KRAS* mutations as the presence of a *KRAS* mutant clone means that the anti-epidermal growth factor receptor (anti-EGFR) antibody cetuximab will be an ineffective treatment ^75^.

**Figure 1.**
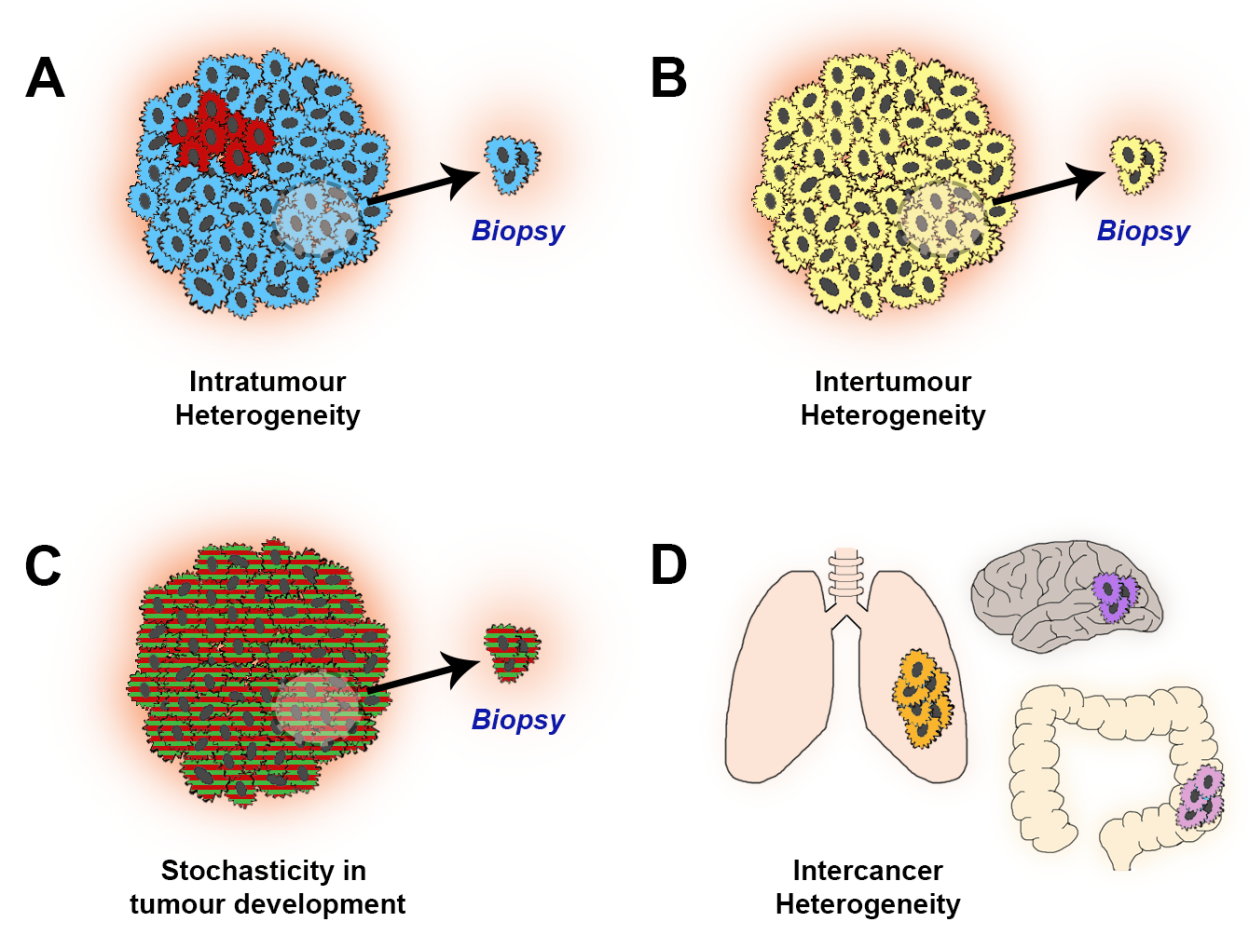
Intra-tumour heterogeneity and prognostication. ( **A**) Intra-tumour heterogeneity means a targeted biopsy may miss a lesion with a poor prognostic signature (“red” phenotype) within the tumour (majority of tumour has a “blue” phenotype that is associated with a good prognosis). ( **B**) Differences between tumours (inter-tumour heterogeneity) mean that a single prognostic biomarker may be unsuitable for use in some tumours that have evolved along a different carcinogenic pathway; this yellow tumour does contain either the previously identified good (blue) or bad (red) phenotypes. ( **C**) Epistasis between genes (or other intra- or inter-cellular interactions) can alter the prognostic value of any individual feature; here, the presence of the “green” mutation may alter the bad prognosis of the “red” mutation assayed in ( **A**). ( **D**) Inter-cancer heterogeneity means that a feature-based prognostic marker developed for one cancer type is unlikely to work in another cancer type.

Clearly, ITH presents a major obstacle for such “feature-based” prognostic markers, simply because if the feature being assayed for is not present in the particular biopsy analysed but is present elsewhere in the tumour then prognosis will be incorrectly assigned ( Figure 1A). A striking demonstration of this was provided by Gerlinger and colleagues when they compared gene expression-based prognostic signatures derived from spatially distinct regions on a single renal cancer and reported that the different regions of the same tumour can harbour either good- or bad-prognosis signatures; therefore, a single biopsy would not sufficiently represent the tumour composition ^8^. Epistatic interactions (perhaps driven by unmeasured genes) could also potentially alter the prognostic value of individual molecular feature ( Figure 1C). There is also an issue of what feature (or indeed set of features) to include in a prognostic assay. One of the striking findings of the recent large-scale extensive molecular characterisations of tumours, such as The Cancer Genome Atlas project ( http://cancergenome.nih.gov), is that every tumour appears molecularly unique or, in other words, that there appear to be many different ways to produce a particular cancer. This rampant *inter-*tumour heterogeneity may preclude a “one size fits all” approach to feature-based prognostic markers since a particular prognostic feature is unlikely to work for all cancers of a particular type ( Figure 1B). Relatedly, the differences between cancers of distinct types may mean that a particular feature is unlikely to have broad prognostic value ( Figure 1D), although there is evidence suggesting that integral biochemical cellular features such as the ability to form proper DNA segregation machinery ^77, 78^ or to properly regulate adhesion ^79^ may have potential in this respect.

The idea that some molecular changes may be integral to tumour biology, and so not subject to ITH, is supported by an intriguing study of renal cancers that provides a counterpoint to the findings of Gerlinger and colleagues. Rini and colleagues used a large cohort of renal cancers to derive a 16-gene signature that predicted recurrence ^76^. Importantly, the authors also performed multi-region sampling on a small number of samples to demonstrate little or no ITH in their gene signature, which, counter to the findings of Gerlinger and colleagues, would suggest that sampling one biopsy can in fact be enough to infer prognosis. In broad terms, the prognostic importance of ITH is likely to be dependent on many factors such as tumour type, tumour stage, and treatment regime, and critically the nature of the biomarker itself.

**Figure 2.**
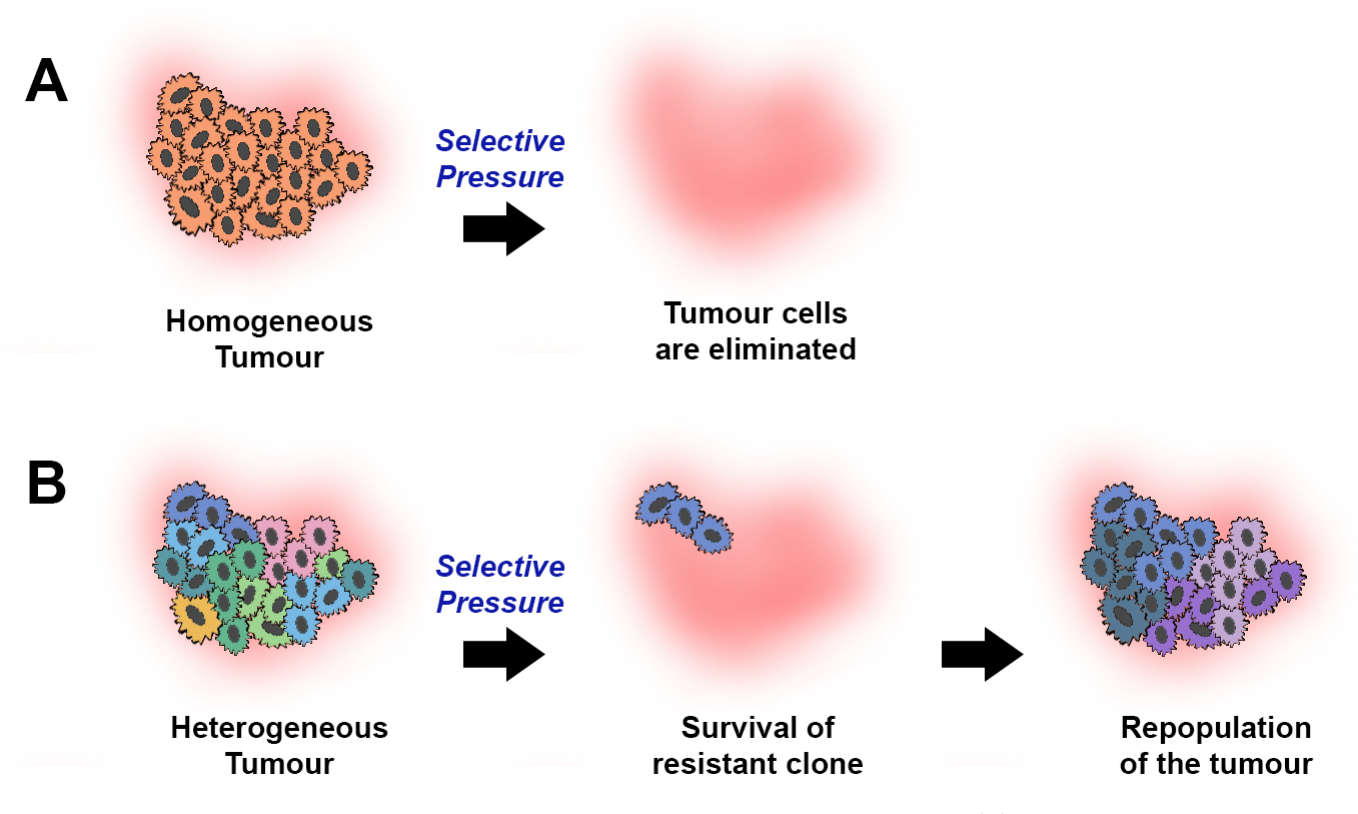
Intra-tumour diversity as a universal prognostic marker. A homogeneous tumour ( **A**) will be eradicated in response to a selective pressure such as chemotherapy, whereas a heterogeneous tumour ( **B**) is more likely to contain a pre-existing resistant clone that survives the selective pressure and seeds the repopulation of the tumour.

However, it is not all doom and gloom. Approaching ITH from an evolutionary perspective suggests a new approach to prognostication that exploits ITH rather being hindered by it. The idea is that the *evolvability* of a population is determined (somewhat) by the degree of diversity present in that population ( Figure 2). To understand this idea, suppose there are two populations of cells: one where all the cells are identical to one another (low diversity) and one where all the cells are phenotypically distinct (high diversity). If the cells in the first scenario experience a new selective pressure (e.g. a cytotoxic drug), then either all the cells are perfectly adapted and nothing happens, or the population is eradicated. When the same selective pressure is applied in the second scenario, the sensitive cells in the phenotypically diverse population will be killed off and any (perhaps minor) subclone that was resistant to the pressure will survive and grow to dominate the tumour in the absence of competing clones. Therefore the idea is that more diverse tumours are more likely to be more evolvable and hence more likely to generate a metastatic clone or contain a clone that is resistant to therapy (or both): hence, the theory is that tumours with higher levels of ITH should have a worse prognosis.

Empirical studies support the idea of quantification of ITH as a prognostic biomarker. Originally, measurements of intra-lesion genetic diversity within the premalignant condition Barrett’s oesophagus were found to be strong predictors of cancer development risk ^40^, and the prognostic value of the diversity signal appeared largely robust to the statistic used to quantify it ^80^. Clonal diversity has also been found to be higher in breast cancer subtypes associated with a worse prognosis ^30^ and is associated with worse overall survival in head and neck cancers ^81^, acute myeloid leukaemia ^82^, ovarian cancers ^37^, and lung cancers ^11^. Importantly, the prognostic value of ITH measures does not appear to be limited to measure of *genetic* heterogeneity, since quantification of morphological heterogeneity in the organisation of tumour and stromal cells in breast cancers ^83^ and the ITH of positron emission tomography-computed tomography signal in lung cancers ^84^ has also been reported to significantly correlate with outcome. These studies give support to the idea that quantification of ITH, as a measure of the carcinogenic process itself rather than a specific feature of that process, may be a *universal prognostic biomarker* suitable for use in all cancer types. Further work is required to determine which features of tumour biology should be assessed for heterogeneousness in order to best determine prognosis. For example, much genetic ITH may be irrelevant for tumour biology (as discussed above) and so naive genetic ITH measures may have limited prognostic value.

Intriguingly, tumours that have underlying defects in DNA repair (such as mismatch repair ^85, 86^ or polymerase-epsilon ^87^ defective tumours of the colon), which are expected to have very high levels of genetic ITH, paradoxically have very good prognoses ^88– 90^. Similarly, with an expression-based signature to quantify the degree of chromosomal instability (CIN) in breast cancers, a measure which likely correlates with ITH, the patients with the worst outcome were those whose tumours had intermediate levels of CIN, whereas patients whose tumours had very high levels of CIN had better outcomes ^91^. One possible explanation for these data is that very high mutation rates, while generating lots of diversity that makes the tumour more evolvable, also generate lots of deleterious variants that impede clone growth. A further contributing factor to this favourable prognosis (at least in tumours with an elevated point mutation rate, such as microsatellite unstable tumours) is the elevated immunogenicity of tumours with a high mutation rate, a feature that is the result of the generation of many immunogenic neo-antigens, which stimulate the host immune system ^92^. Effective ITH-based prognostic markers will need to address such complexities of tumour evolution to be broadly useful. We note too that existing broad molecular classifications of cancer (such as microsatellite versus chromosomally unstable cancers in the colon) can have significant prognostic value themselves, and consequently ITH-based measures may be useful only to stratify within such molecularly defined subgroups.

## Intra-tumour heterogeneity and treatment

Resistance to chemo- and targeted-therapy, and concomitant treatment failure, occurs in the majority of cases ^72, 93– 97^. Pre-existing ITH can be (indirectly) attributed as the underlying cause of these treatment failures. Tumours, at the stage when they are treated, contain many billions of cells and it appears an almost mathematical certainty that at least a few of these cells will have evolved a therapy-resistant phenotype. Indeed, in patients with colorectal cancer treated with the anti-EGFR antibody cetuximab, empirical measurements show that KRAS-mutant clones that likely existed at undetectably low frequency prior to the initiation of therapy expand exponentially at the administration of therapy ^98^ and similar dynamics have been observed in a variety of other malignancies, including lung ^99, 100^, leukaemia ^23, 101^, and melanoma ^102, 103^.

Is ITH an insurmountable barrier to effective cancer treatment? It is clearly a major challenge, and accordingly one approach that has been suggested is to try to suppress the level of ITH within a tumour (by targeting the drivers of genetic instability) in order to provide a more homogenous tumour that may be more pliable with treatment ^104^. An exciting but radically different approach is to try to use the presence of ITH itself in order to increase the efficacy of cancer treatment ^105^. The approach, termed *adaptive therapy*, is based on the maxim that “nothing comes for free”. This is the idea that resistance to a particular therapeutic agent inevitably carries some cost to the cancer cell, so that in the *absence* of therapy a resistant clone is at a disadvantage and so will be outcompeted by any remaining sensitive cells. The adaptive therapy approach is therefore to pulse the drug in such a manner that when the drug is present the sensitive cell population is killed off and the resistant cell population prospers, whereas when the drug is absent the resistant population is outcompeted by the sensitive population. In theory, if the sensitive population is able to outcompete the resistant population sufficiently well, this treatment regime will mean that the tumour remains sensitised to the drug over long time frames while the size of tumour population as a whole is effectively constrained ^106, 107^. In a melanoma xenograft model, this idea appears efficacious ^108^. Translation of this idea to the clinic would require the development of appropriate monitoring tools that indicate when to provide/withdraw treatment; this is clearly a major challenge in itself. It is important to note that the idea behind adaptive therapy is not to affect a cancer cure—indeed, the idea is predicated on the assumption that this is impossible with a single agent—but rather to extend the effectiveness of a particular agent indefinitely and in so doing make the cancer a chronic rather than fatal disease. **

Other approaches have been proposed that similarly attempt to *steer* the evolutionary response of a tumour to treatment ^109, 110^. The general idea of these innovative proposals is that it is possible to predict which phenotypes will emerge from a heterogeneous population under treatment, and so the next therapy can be applied in order to treat the emergent clone. This logic provides the rationale behind attempts to “vertically” combine targeted therapies (targeting multiple members of the same pathway) to prevent resistance to a single agent emerging because of the selective pressure the mono-agent provides for the emergence of a clone with a mutation in a gene downstream of the original drug target ^111, 112^. In general, combination therapies may also help to tackle the issue of pre-existing resistance in heterogeneous tumours, since the chance of a cell being doubly resistant to two different therapies should be proportional to the probability that the cell is resistant to any one therapy individually, and so if mono-therapy resistance is rare in the tumour cell population, then doubly resistant cells should be vanishingly rare indeed ^113^. An emerging theme of combination therapy is the use of both targeted and non-specific therapies, such as immunotherapy. For example, a combination of BRAF and MEK inhibitors with adoptive cell transfer (ACT) immunotherapy in a BRAF-driven mouse model of melanoma has been shown to induce complete tumour regression ^114^. However, it should be noted that combining multiple therapies may carry the cost of increased toxicity to normal tissue. Together, these data and theoretical studies suggest that therapeutic regimes will need to be *personalised for the evolutionary response of an individual tumour* in order to effectively tackle the problem of ITH.

## Conclusions

ITH is an inevitable feature of all cancers and presents challenges to our understanding of tumour biology and our ability to prognose and treat cancer. But it is our opinion that these challenges are not insurmountable, and in fact understanding the processes generating ITH provides us with a window to understand the very drivers of carcinogenesis itself. From a more clinical perspective, quantification of ITH offers an exciting opportunity to improve prognostication, and exploiting rather than ignoring ITH has the potential to improve the efficacy of existing cancer therapies.
